# Ebullition of oxygen from seagrasses under supersaturated conditions

**DOI:** 10.1002/lno.11299

**Published:** 2019-08-08

**Authors:** Matthew H. Long, Kevin Sutherland, Scott D. Wankel, David J. Burdige, Richard C. Zimmerman

**Affiliations:** ^1^ Marine Chemistry and Geochemistry Department Woods Hole Oceanographic Institution Woods Hole Massachusetts; ^2^ Department of Earth, Atmospheric and Planetary Sciences Massachusetts Institute of Technology Cambridge Massachusetts; ^3^ Department of Ocean, Earth and Atmospheric Sciences Old Dominion University Norfolk Virginia

## Abstract

Gas ebullition from aquatic systems to the atmosphere represents a potentially important fraction of primary production that goes unquantified by measurements of dissolved gas concentrations. Although gas ebullition from photosynthetic surfaces has often been observed, it is rarely quantified. The resulting underestimation of photosynthetic activity may significantly bias the determination of ecosystem trophic status and estimated rates of biogeochemical cycling from in situ measures of dissolved oxygen. Here, we quantified gas ebullition rates in *Zostera marina* meadows in Virginia, U.S.A. using simple funnel traps and analyzed the oxygen concentration and isotopic composition of the captured gas. Maximum hourly rates of oxygen ebullition (3.0 mmol oxygen m^−2^ h^−1^) were observed during the coincidence of high irradiance and low tides, particularly in the afternoon when oxygen and temperature maxima occurred. The daily ebullition fluxes (up to 11 mmol oxygen m^−2^ d^−1^) were roughly equivalent to net primary production rates determined from dissolved oxygen measurements indicating that bubble ebullition can represent a major component of primary production that is not commonly included in ecosystem‐scale estimates. Oxygen content comprised 20–40% of the captured bubble gas volume and correlated negatively with its δ^18^O values, consistent with a predominance of mixing between the higher δ^18^O of atmospheric oxygen in equilibrium with seawater and the lower δ^18^O of oxygen derived from photosynthesis. Thus, future studies interested in the metabolism of highly productive, shallow water ecosystems, and particularly those measuring in situ oxygen flux, should not ignore the bubble formation and ebullition processes described here.

The formation of gas bubbles on photosynthetic surfaces occurs commonly during periods of high productivity in many aquatic ecosystems (Jørgensen et al. 1979; Revsbech and Jorgensen 1983). Bubbles have been reported on the surface of sediments on sunny days during high oxygen (O_2_) saturation (Hunding and Hargrave 1973), on algal communities on the bottom surface of the ice during low flow and high irradiance conditions (Ashworth and Ryan 2000), and especially on freshwater macrophytes (Reinke 1883; Blackman and Smith 1911; Wilmott 1921; Odum 1957) and marine seagrass leaves (Drifmeyer 1980; Roberts and Caperon 1986; Wilson et al. 2012). The direct ebullition of bubbles from seagrass has been observed in situ, occurring during high irradiance conditions and high O_2_ concentrations. In fact, previous studies have described ebullition from seagrass beds as similar to a “newly opened bottle of beer” (Zieman 1974) or “minute streams of oxygen bubbles” emanating from seagrass leaves (Hargraves 1982).

Early experimental work used bubble ebullition from cut aquatic plants to measure carbon dioxide assimilation and photosynthetic rates in the laboratory (Reinke 1883; Blackman and Smith 1911). Later improvements produced “Wilmot's Bubbler,” which allowed for more accurate measurements of ebullition and identified several difficulties associated with estimating ebullition including variations in bubble gas concentrations, bubble size, pressure, and gas saturation states (Wilmott 1921). These studies revealed that the combined influence of physical bubble properties (e.g., the ideal gas law) and natural biological variability (e.g., photosynthetic biomass and light response, photorespiration) complicated in situ quantification of ebullition in natural aquatic systems.

While the ebullition of O_2_ gas from photosynthetic systems has been observed, dissolved O_2_ is most commonly used to estimate in situ rates of aquatic photosynthesis, respiration, and net ecosystem metabolism (Odum 1957; Middelburg et al. 2005; Glud 2008). Oxygen is ~ 30 times less soluble in seawater than carbon dioxide, another potential tracer of photosynthesis, and O_2_ concentrations are independent of seawater pH as it does not undergo acid‐base speciation. These traits make O_2_ an advantageous tracer of aquatic metabolism as its production or consumption is easily measured by dissolved O_2_ sensors. However, low O_2_ solubility often leads to wide variations in saturation state, particularly in low volume systems that have high rates of primary production and respiration. Such large shifts in saturation state often result in the formation of bubbles. The direct flux of O_2_ transported in bubbles to the atmosphere therefore results in an underestimation of photosynthetic rates as determined from measurements of changes in dissolved O_2_ over time (Jørgensen et al. 1979; Revsbech and Jorgensen 1983; Borum et al. 2007).

Although substantial qualitative evidence exists for the formation and ebullition of bubbles in photosynthetic systems, little attention has been applied to quantifying seagrass oxygen ebullition rates and the in situ conditions that promote bubble formation. Acoustic seagrass mapping and detection of free bubbles has been explored as a tool for estimating photosynthesis (Wilson and Dunton 2009; Wilson et al. 2010, 2012; Felisberto et al. 2015), optical detectors have been used to quantify bubble spatiotemporal distribution and size (Delwiche and Hemond 2017a,*b*), and models using noble gas concentrations have been developed to estimate ebullition rates (Howard et al. 2018). However, these methods have not conducted direct measurements of bubble O_2_ content, which is needed to accurately determine the fraction of total photosynthetic O_2_ released as bubbles. Therefore, a quantification of photosynthetic O_2_ production by bubble ebullition methods requires independent quantification of bubble O_2_ concentration to determine the fate of photosynthetic O_2_ in shallow surface waters.

Inverted funnel traps have been used to evaluate bubble ebullition and composition, primarily to investigate microbial production of methane, nitrous oxide, nitrogen, and carbon dioxide in freshwater systems (Keller and Stallard 1994; Casper et al. 2000; Huttunen et al. 2001; Varadharajan et al. 2010; Gao et al. 2013). More recently, these techniques have been used to investigate O_2_ release from sediments and microphytobenthic communities (Cheng et al. 2014; Koschorreck et al. 2017). The simplest design employs an inverted funnel to capture gas, followed by manual collection and measurement of the gas volume (Odum 1957; Martens and Klump 1980; Keller and Stallard 1994; Cheng et al. 2014; Koschorreck et al. 2017). These bubble traps can be easily deployed to quantify seagrass ebullition as conditions favorable for bubble formation have been identified (e.g., shallow water, high irradiance, low flow, high oxygen saturation) (Zieman 1974; Hargraves 1982).

In this study, we used bubble traps to quantify rates of seagrass ebullition and the subsequent direct flux of gas to the atmosphere. Ebullition was measured over shallow *Zostera marina* seagrass meadows in Virginia, U.S.A. at two sites where intense sampling was conducted during conditions likely to favor ebullition. Our goals were to (1) explore the relationship between rates of bubble formation, irradiance, and tidal stage that controls the overlying water depth, (2) evaluate the isotopic composition (as δ^18^O) and concentration of O_2_ in the bubble gas to quantify the photosynthetic source of O_2_ and to estimate rates of bubble equilibration with the surrounding seawater, (3) estimate the transport of photosynthetically derived O_2_ directly to the atmosphere via bubbles relative to rates of O_2_ flux derived from dissolved O_2_ measurements, and (4) characterize the temporal relationship between the timing of photosynthetic O_2_ production and vertical transport of bubbles to the sea surface.

## *Methods*

### Study sites

Photosynthetic bubble formation was investigated in two eelgrass (*Z. marina* L.) meadows in shallow coastal bays (Spider Crab Bay: 37.342617°N, −75.802853°W and South Bay: 37.272783°N, −75.806097°W) on the Virginia portion of the Atlantic side of the DelMarVa Peninsula, U.S.A. The meadows extended for hundreds of meters from both measurement locations in all lateral directions. The sites were within 100 m of the Virginia Institute of Marine Sciences water quality stations (https://stormcentral.waterlog.com/public/vims) where water column O_2_, temperature, salinity, and depth were monitored every 15 min using a factory‐calibrated EXO2 sonde with accuracies of ± 1% O_2_, ± 0.01°C, ± 0.1 salinity, ± 0.004 m, respectively. Photosynthetically active radiation (PAR) was measured at each site using an Odyssey PAR logger (Odyssey, NZ) which was calibrated, in situ by the methods of Long et al. (2012) to a factory‐calibrated 4‐channel HR4 spectroradiometer system (HOBI Labs HydroRAD‐4).

### Seagrass characteristics

**Figure 1:**
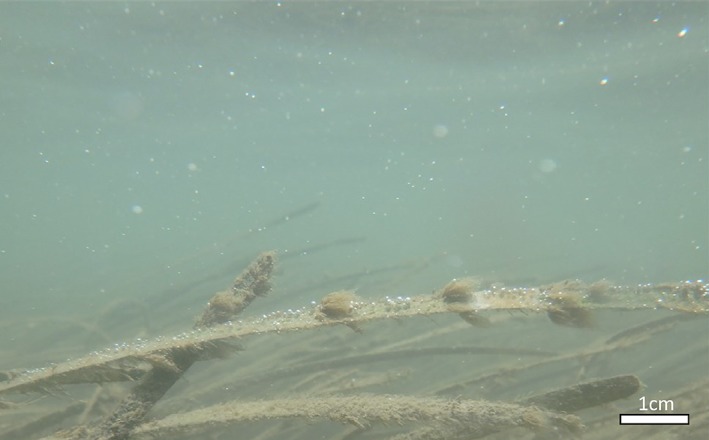
Image of O_2_ ebullition from *Z. marina* seagrass bed in South Bay, Virginia on 20 July 2017 at 12:45 h (*see* Fig. 2). Note the presence of bubbles on the upper leaf surface of a relatively clean leaf and in the water column above, but not on the heavily fouled leaves or clumps of periphyton and filamentous algae.

Seagrass density at both locations was quantified by counting all shoots in twenty to thirty 0.05 m^2^ quadrats randomly located within a 20 m radius of the bubble traps. One shoot was harvested from each quadrat for determination of leaf size‐frequency distribution, and leaf area index (LAI). Leaves were cleaned of periphyton, as our visual observations indicated that most bubbles were forming on relatively clean younger leaves, and not the heavily fouled older leaves or the associated periphyton (Fig. 1), by gentle scraping with a razor blade (Fig. 1). The clean shoots were dried at 60°C for 14 d and weighed on a top‐loading balance (precision 0.001 g).

### Bubble traps

Four to eight bubble traps where deployed, each 3 m apart, in July 2017 at the two sites over 4 d in Spider Crab Bay and over 5 d in South Bay. The bubble traps consisted of an inverted funnel (maximum diameter = 30 cm, overall height = 25 cm) constructed from a metal ring, a clear vinyl skirt and 13 cm diameter (wide end) plastic laboratory funnel. The clear vinyl was cut into a cone shape and sealed inside the smaller funnel with silicon glue and rivets (Supporting Information Fig. S1). A plastic stopcock with a Luer‐Lok™ tip was mounted to the narrow end of the plastic funnel. The buoyant funnel was anchored to the bottom by four adjustable lines, one from each quadrant of the metal ring such that the stopcock was level with the water surface.

Each trap was submerged, cleaned of any debris, and cleared of all bubbles each day. The traps were sampled approximately hourly when on‐site during the daytime, with longer sampling intervals occurring overnight (*see* Supporting Information). The captured gas was collected through the stopcock at the top of the funnel‐mounted syringe using a glass, gas‐tight syringe with the volume determined by the syringe draw required to remove all headspace gas from the trap. The gas volume was recorded and, for a subset of samples (*see* Supporting Information Table S1), a 2 mL aliquot of the sampled gas was injected into an evacuated 2 mL glass crimp vial. Oxygen content of the 2 mL aliquots of bubble trap gas was measured using a fixed‐needle O_2_ optode and meter (Pyroscience, GE) (Koschorreck et al. 2017). The optode was calibrated using high‐purity nitrogen gas (Airgas, 99.99%) and aviation grade O_2_ gas (Airgas, 99.95%) by injecting aliquots of each gas into 2 mL crimp vials to produce O_2_ standards (2 mL each) of 0%, 25%, 50%, and 100% O_2_. Standards, blanks (no gas added), and gas samples were analyzed by piercing the vial septa and allowing the optode to equilibrate.

### Oxygen isotopes

Oxygen isotope ratios and O_2_:Ar ratios were measured with a multicollector IsoPrime100 isotope ratio mass spectrometer coupled to a gas chromatograph with a manual injection port (Sutherland et al. 2018). The same 2 mL crimp vials samples, which were previously evaluated by the O_2_ optode, were used for this analysis as the optical measurements did not require modification of the gas sample. Prior to analysis, samples were slightly pressurized with an aliquot of high purity helium to prevent the sample from mixing with air while sampling. The sample was introduced to the injection port using a helium flushed, gas‐tight syringe. Downstream of the injection port the sample was passed through a 2 m molecular sieve (5 å) gas chromatography column (Restek; OD 1/16″) for separation of O_2_ and Ar from N_2_. Moisture was removed from each sample by a 2 m Nafion dryer with a dry, helium‐purged jacket (Permapure). Signal intensities for mass/charge (*m*/*z*) ratios of 32, 34, and 40 were monitored simultaneously to determine the O_2_ isotope ratio and O_2_:Ar, and *m*/*z* of 28 and 29 were used to monitor N_2_ and validate sample integrity. Oxygen isotopic compositions were expressed using delta notation with δ^18^O values in units of per mil (‰) with respect to Vienna Standard Mean Ocean Water (VSMOW). Oxygen isotope measurements were standardized to lab air, taken as +23.88‰ (Barkan and Luz 2005). All O_2_:Ar measurements were standardized to dissolved gas taken from water in equilibrium with lab air at room temperature, which was collected after introducing a helium headspace above the water in a sealed serum vial and shaking for a minimum of 30 min. Oxygen concentration measurements were derived from sample O_2_:Ar ratio. Generally speaking, the oxygen fraction of each sample is as follows:

$$X_{O_{2}}=\frac{\frac{\left(n_{O_{2}-\mathrm{eq}}+n_{O_{2}-\text{photo}}\right)}{n_{\mathrm{Ar}}}}{\frac{\left(n_{O_{2}-\mathrm{eq}}+n_{O_{2}-\text{photo}}+n_{\text{Other}}\right)}{n_{\mathrm{Ar}}}}$$

where *n* represents the number of moles of each gas including O_2_, Ar, and all other gases. If we make the simplifying assumption that all dissolved gases diffused into the bubble at approximately the same rate and the sources of dissolved gas are either atmospheric or photosynthetic, we can solve for $X_{O_{2}}$ as a function of the observed O_2_:Ar ratios:

$$X_{O_{2}}=\frac{\left[{\left(\frac{n_{O_{2}}}{n_{\mathrm{Ar}}}\right)}_{\text{sample}}/{\left(\frac{n_{O_{2}-\mathrm{eq}}}{n_{\mathrm{Ar}}}\right)}_{\mathrm{eq}}\right]}{\left[{\left(\frac{n_{O_{2}}}{n_{\mathrm{Ar}}}\right)}_{\text{sample}}/{\left(\frac{n_{O_{2}-\mathrm{eq}}}{n_{\mathrm{Ar}}}\right)}_{\mathrm{eq}}\right]+\frac{0.792}{0.208}}$$

Reproducibility of δ^18^O and O_2_:Ar for lab air standards in this study were 0.06‰ and 0.2%, respectively (1 standard deviation, *n* = 18). In the theoretical treatment of this equation, we assume the end‐members of 24.5‰ and 0‰ for dissolved oxygen in equilibrium with seawater and photosynthetic O_2_, respectively (Benson and Krause 1984; Luz and Barkan 2000; Barkan and Luz 2005). It is important to note that water may undergo some oxygen isotope fractionation during photosynthetic O_2_ production (Luz and Barkan 2011a). Photosynthetic O_2_ was observed to range from ~ 0‰ for cyanobacteria to as high as ~ 6‰ in some eukaryotic algae. This effect is small relative to the magnitude of the difference between photosynthetic oxygen and atmospheric O_2_, and thus δ^18^O of dissolved oxygen is useful for fingerprinting the addition of isotopically light photosynthetic O_2_ into the system. We also note that this mixing construction does not consider the influence of respiration on δ^18^O, and therefore cannot be strictly used for interpretation of a two end‐member mixture. Qualitatively, respiration will decrease the O_2_ concentration, and enrich the residual O_2_ in ^18^O (Luz and Barkan 2011b), thereby dampening the dynamic range of a mixture between atmosphere and photosynthetic O_2_. As such, we note that δ^18^O alone is insufficient to quantitatively disentangle these three processes.

## *Results*

Extensive eelgrass meadows were present at both sites, with mean biomass density two times higher in South Bay (315 ± 56 g dry weight m^−2^ [gDW m^−2^]) than in Spider Crab Bay (163 ± 40 gDW m^−2^) (Table 1). The leaf biomass was 197 ± 46 g DW m^−2^ and 106 ± 26 g DW m^−2^ and the LAI was 3.25 ± 0.37 and 1.82 ± 0.19 for South Bay and Spider Crab Bay, respectively. Both sites are heavily influenced by tidal exchange with the open waters of the Mid‐Atlantic Bight, as indicated by large variations in water depth, temperature, and O_2_ saturation (Table 1). Gas bubble formation on eelgrass leaves and ebullition through the water column were visibly present at both sites at low tide (e.g., Fig. 1).

**Table 1:** Site characteristics and gas fluxes.

	Units	South Bay		Spider Crab Bay	
		Mean ± SD	Range (min–max)	Mean ± SD	Range (min–max)
Standing biomass	gDW m^−2^	315 ± 56	104–433	163 ± 40	63–290
Leaf biomass	gDW m^−2^	197 ± 46	92–321	106 ± 26	35–199
LAI		3.25 ± 0.37	1.28–4.83	1.82 ± 0.19	0.40–3.39
Depth	m	1.20 ± 0.47	0.41–2.09	1.41 ± 0.42	0.56–2.25
Salinity		32.3 ± 0.29	31.4–32.8	32.7 ± 0.23	31.6–33.2
Temperature	°C	27.3 ± 1.27	25.2–31.4	28.2 ± 1.2	24.9–31.3
O_2_	% saturation	95.7 ± 20.6	55.7–186.0	97.8 ± 8.6	83.0–132.1
Gas flux	mmol m^−2^ h^−1^	2.0 ± 2.5	0.0–7.3	0.8 ± 1.6	0.0–4.5
Gas O_2_	%	20.4 ± 13.8	0.0–40.5	12.4 ± 12.2	0.0–29.4
Bubble O_2_ flux	mmol O_2_ m^−2^ h^−1^	0.4 ± 0.6	0.0–3.0	0.1 ± 0.22	0.0–1.3

**Figure 2:**
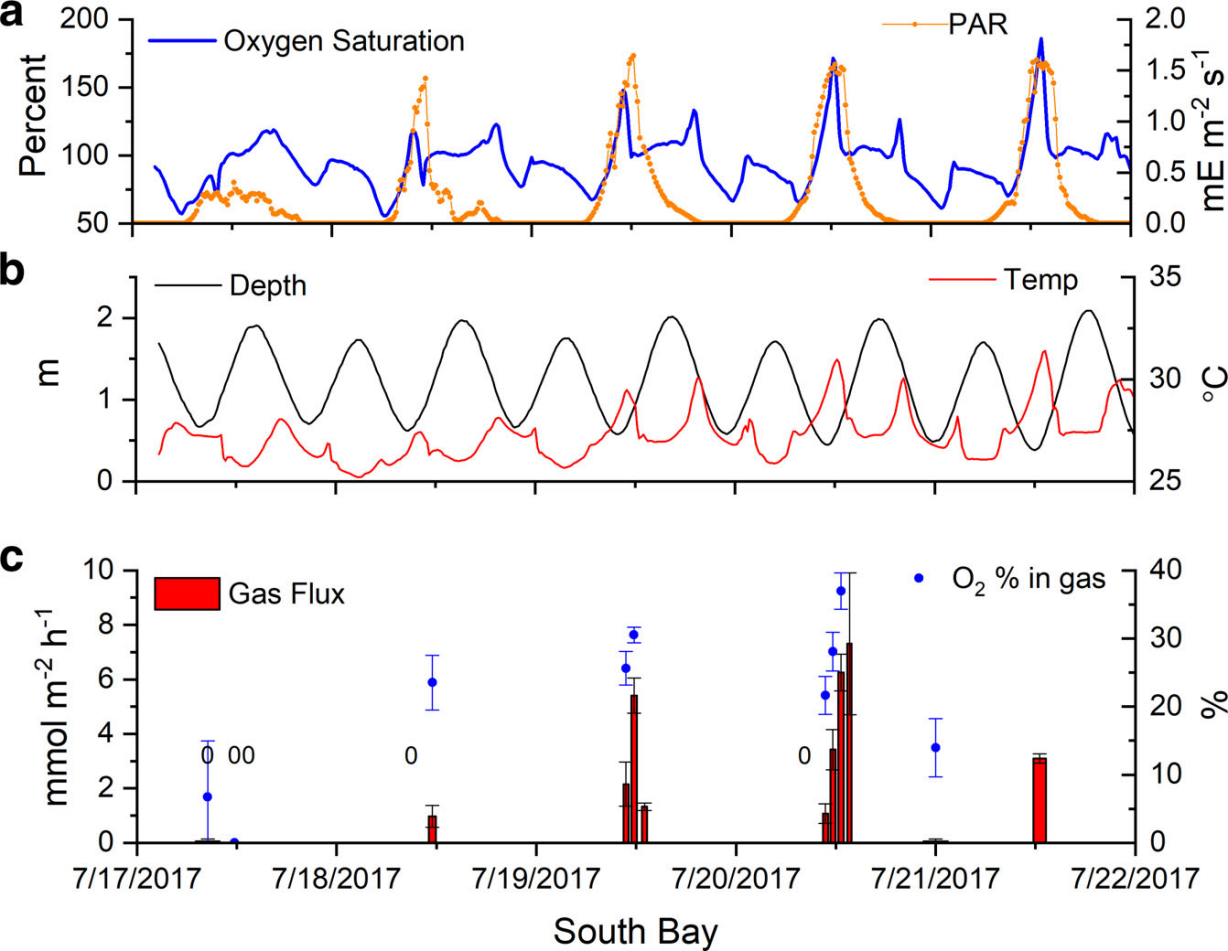
Oxygen percent saturation in South Bay, VA, U.S.A., over 5 d where seagrass ebullition was observed (**a**, left, blue). Broadband irradiance was measured as PAR above the canopy (**a**, right, orange). The average depth (left, black) and temperature (right, red) are shown in (**b**). The mean gas ebullition from the seagrass canopy (**c**, red bars, left) where the width indicates relative trap deployment time and a “0” indicates a flux of < 0.05 mmol m^−2^ h^−1^ was observed, and the mean percent O_2_ of the trapped gas (**c**, blue dots, right) are shown in the bottom panel (error bars represent standard errors). Gas fluxes and percent O_2_ were maximal about 1.5–2 h after low tide.

**Figure 3:**
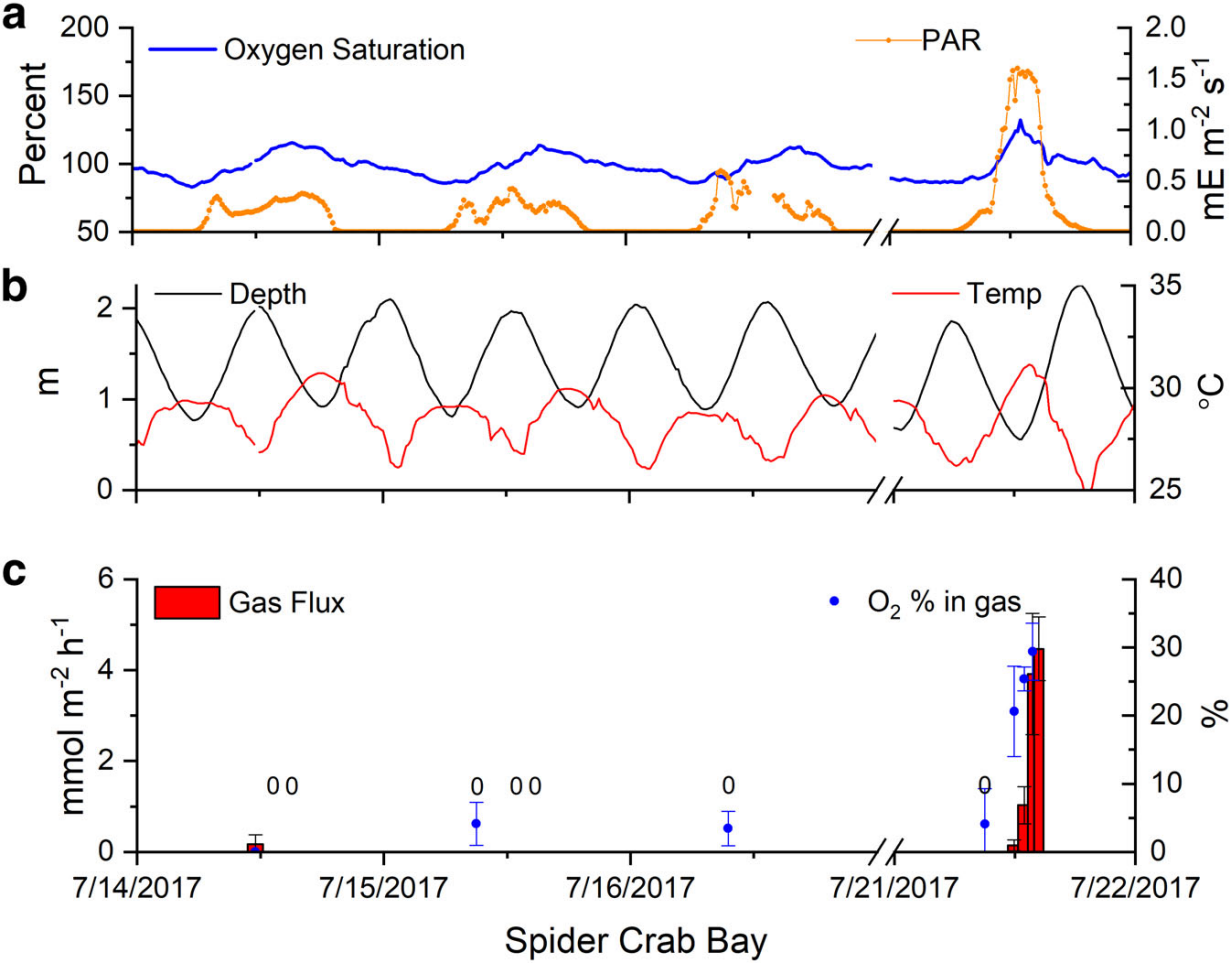
Oxygen percent saturation in Spider Crab Bay, VA, U.S.A., over 4 d where seagrass ebullition was observed (**a**, left, blue). Broadband irradiance was measured as PAR above the canopy (**a**, right, orange). The average depth (left, black) and temperature (right, red) are shown in (**b**). The mean gas ebullition from the seagrass canopy (**c**, red bars, left) where the width indicates relative trap deployment time and a “0” indicates a flux of < 0.05 mmol m^−2^ h^−1^ was observed, and the mean percent O_2_ of the trapped gas (**c**, blue dots, right) are shown in the bottom panel (error bars represent standard errors). Gas fluxes and percent O_2_ were maximal about 1.5–2 h after low tide.

**Figure 4:**
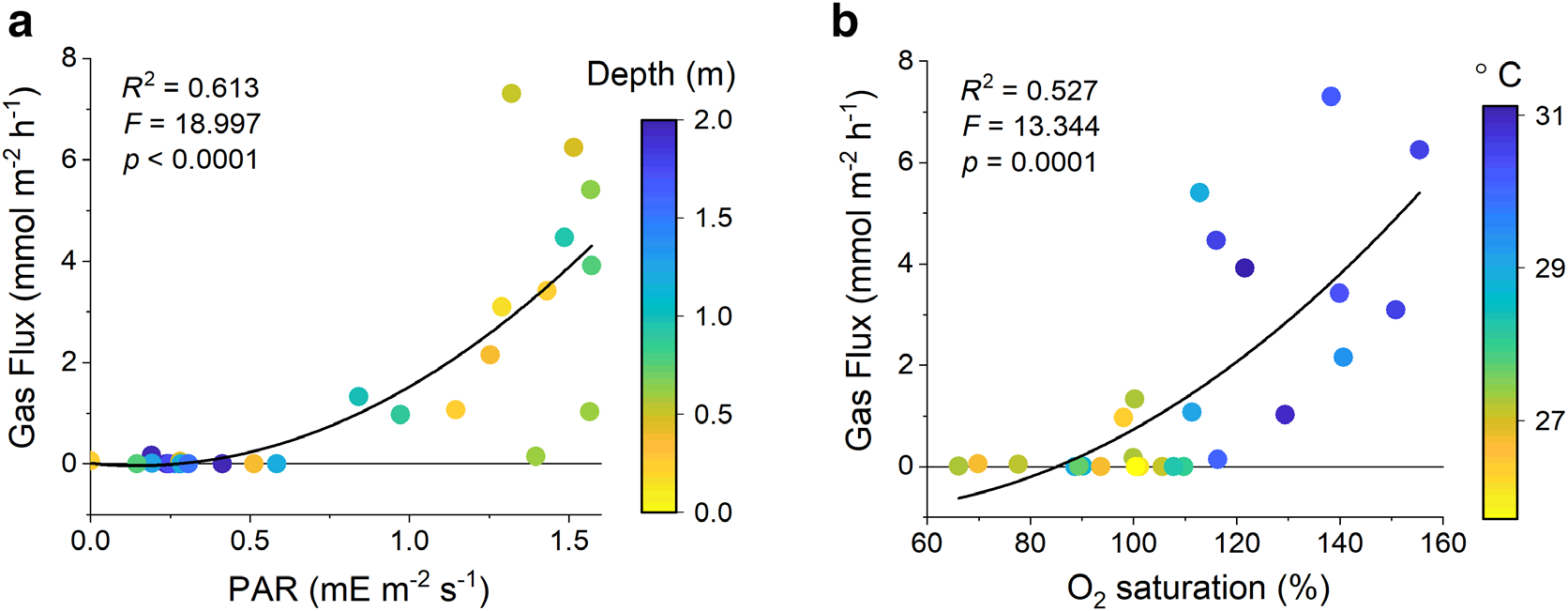
Gas fluxes from both sites plotted vs. water depth and PAR (**a**) and temperature and O_2_ saturation of the water (**b**). High rates of gas ebullition occurred during periods of low water depths and high light (PAR), water O_2_ saturation, and temperature. Second order polynomials were fit to the gas fluxes representing a significantly better fit than a linear regression (*see* Supporting Information for fit parameters).

The flux of gas bubbles (< 0.1 mmol gas m^−2^ h^−1^) and O_2_ concentrations (< 14% O_2_, Figs. 2, 3) were low during overnight periods. Bubble gas fluxes collected during the daytime were much larger (up to 7.3 mmol gas m^−2^ h^−1^) and the captured gas contained higher O_2_ concentrations (up to 41% O_2_) with maxima occurring at the coincidence of low tide and high light conditions. Low rates of gas ebullition were observed (< 1.0 mmol gas m^−2^ h^−1^, Figs. 2, 3) when high tides occurred around the noon period of high irradiance (e.g., 14–18 July). When low tides coincided with high noontime irradiances, maximum gas fluxes were 7.3 mmol gas m^−2^ h^−1^and 4.5 mmol gas m^−2^ h^−1^ with O_2_ concentrations of 41% and 29% at South Bay and Spider Crab Bay, respectively (Table 1). The product of the maximum rates of gas ebullition (7.3 mmol gas m^−2^ h^−1^, Table 1) and the maximum O_2_ concentration of the gas (41% O_2_) yielded a maximum hourly O_2_ flux of 3.0 mmol O_2_ m^−2^ h^−1^ at South Bay. The maximum O_2_ ebullition rates at Spider Crab Bay (1.3 mmol O_2_ m^−2^ h^−1^) were consistent with the lower biomass, LAI, and dissolved gas concentrations (Table 1). The daily gas fluxes, estimated from the summation of hourly fluxes and their mean O_2_ content over each 24 h period, were 0–9.6 mmol O_2_ m^−2^ d^−1^ and 0–10.7 mmol O_2_ m^−2^ d^−1^ for Spider Crab and South Bay, respectively. The gas flux at both sites increased with irradiance, O_2_ saturation, and temperature (Fig. 4). Gas fluxes decreased with increasing depth, likely due to decreased light availability and increased gas solubility and decreased buoyancy with increasing pressure.

**Figure 5:**
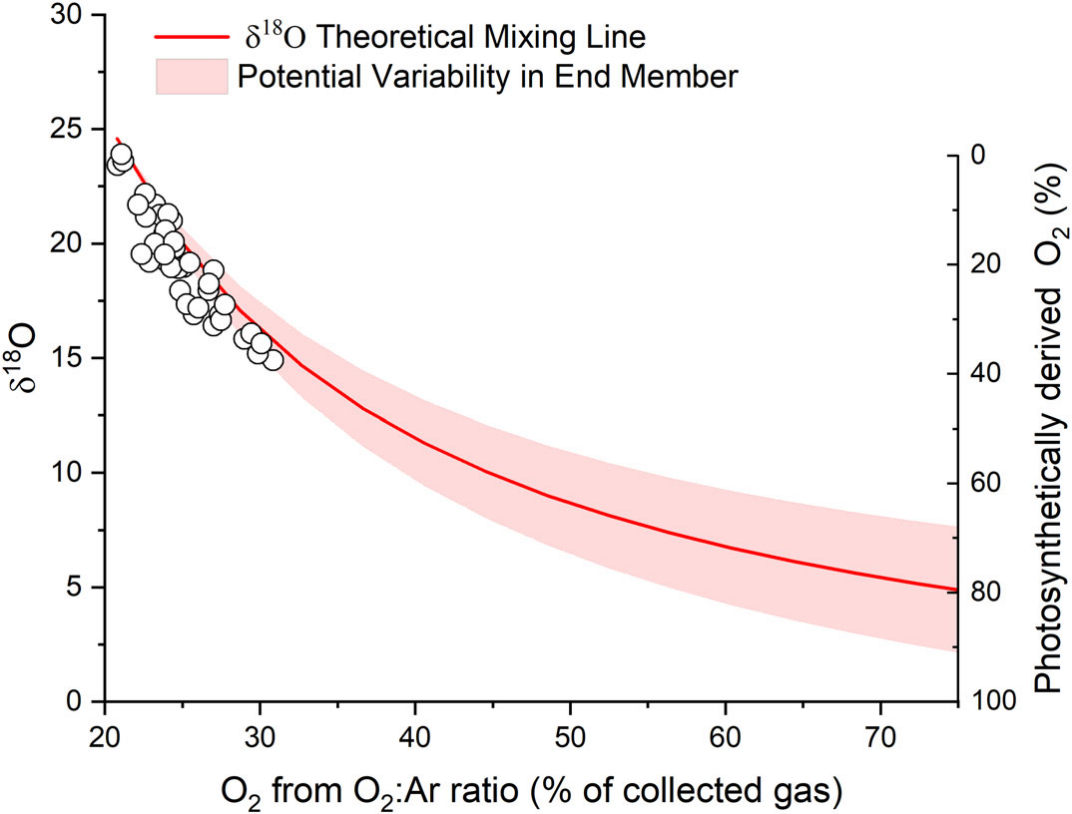
The O_2_ concentration derived from the O_2_:Ar ratio of the sample relative to water in equilibrium with the atmosphere, plotted against δ^18^O value. The isotopic values fall along a theoretical mixing (red) line between atmospheric O_2_ in equilibrium with seawater (δ^18^O = +24.5‰) and that of photosynthetically derived O_2_ (δ^18^O = +3‰ δ^18^O [approx.]). The red shaded region represents the range of possible photosynthetic end‐member sources of ~ 0–6‰ δ^18^O from Luz and Barkan (2011a).

Gas sample concentrations determined from the O_2_ optode and the isotope ratio mass spectrometer were significantly correlated (*see* Supporting Information Fig. S2) and both methods produced a positive relationship between O_2_ gas concentration and ebullition rate. Furthermore, δ^18^O values decreased with increasing O_2_ content of the gas (Supporting Information Fig. S2, Fig. 5). δ^18^O values plot along a theoretical mixing line between atmospheric O_2_ in equilibrium with seawater (δ^18^O ≅ 24.5‰) and that of photosynthetically derived O_2_ (δ^18^O ≅ 0–6‰) (Benson and Krause 1984; Barkan and Luz 2005; Luz and Barkan 2011a). The δ^18^O values suggest that, during periods of low ebullition and bubble O_2_ concentrations near saturation, the gas in the slow‐forming bubbles is in near isotopic equilibrium with the surrounding water (≅ 24.5‰) (Fig. 5). While a slight mismatch between the theoretical mixing line and our δ^18^O data may reflect some influence by respiration and/or isotope effects associated with mass transfer of gas between dissolved and gas phases (Knox et al. 1992), the negative relationship between δ^18^O and concentrations of O_2_ in the collected gas further suggests that O_2_ ebullition rates were directly correlated with photosynthetic production. The influence of respiratory consumption of O_2_ notwithstanding, this simple two‐end‐point mixing model indicates that photosynthetically derived O_2_ transported via bubbles ranged from 2.7% of the evolved gas at the lowest measured O_2_ concentrations to 39.4% of the evolved gas at the highest measured O_2_ concentrations (Fig. 5).

**Figure 6:**
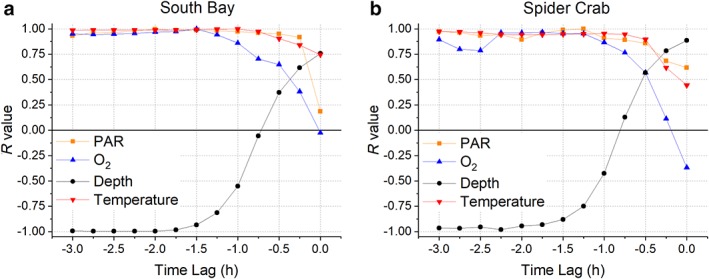
Correlation coefficients (*R* value) for the gas fluxes, during periods of intense sampling on 20 June and 21 June, at South Bay (**a**) and Spider Crab Bay (**b**) plotted against the time‐lagged environmental conditions of PAR, oxygen, depth, and temperature. Gas fluxes where autoregressive with maximum correlation coefficients occurring after ~ 1.5–2 h (*see* Supporting Information Table S2 for detailed statistics).

At both sites, there was an apparent time lag between the optimal conditions for bubble production and when gas bubbles were captured within the traps (Fig. 6). Plotting measured gas fluxes (during periods of intense sampling on 20 June and 21 June) vs. water depth, irradiance, O_2_ saturation, or temperature with a range of time lags for these latter quantities showed that an expected and significant linear relationship between gas flux and these quantities was observed with a time lag of at least ~ 1.5–2 h between observed environmental conditions (water depth, irradiance, O_2_ saturation, or temperature) and measured gas fluxes.

**Figure 7:**
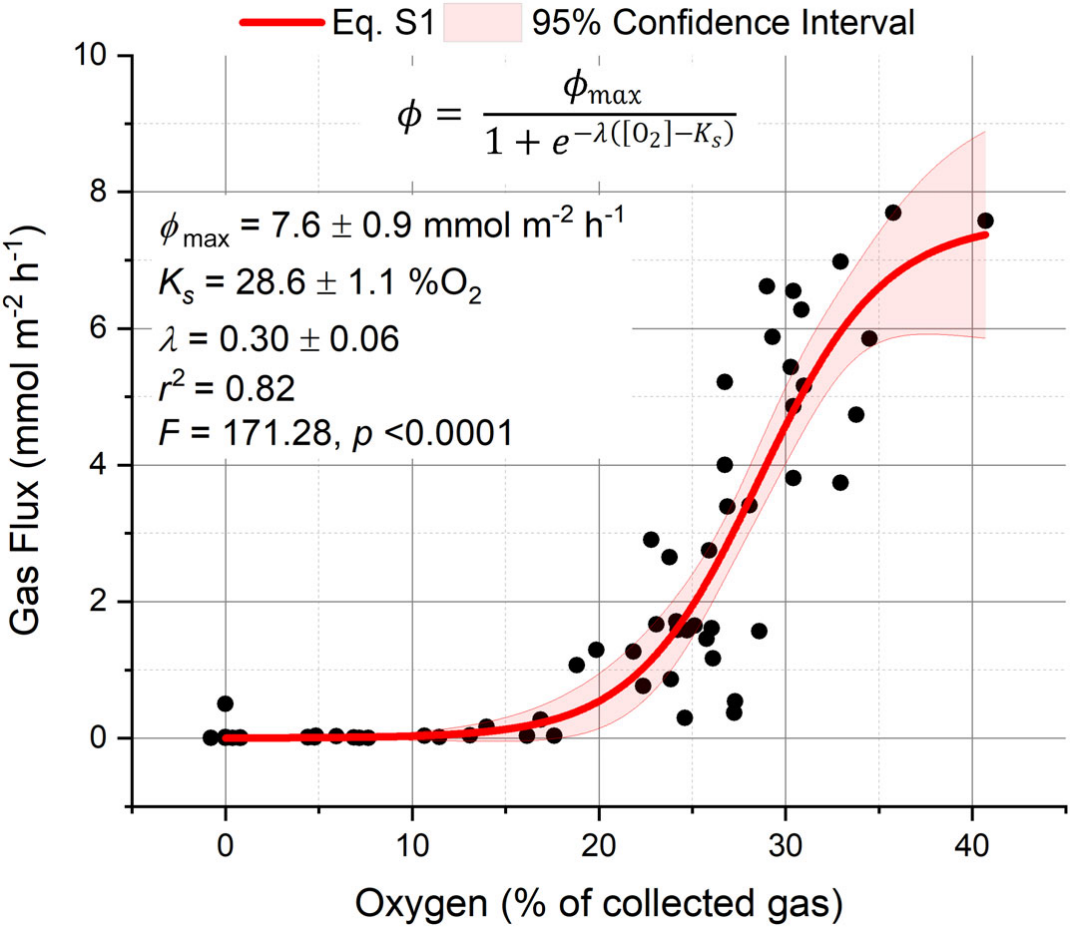
Measured gas flux (*ϕ*) and measured O_2_ percent from the optode from both sites. The optode O_2_ and gas flux measurements were fit to a logistic regression to illustrate the relationship between O_2_ concentration and the initiation of bubble production and the gas fluxes. Supporting Information Eq. S1 (red lines) and the 95% confidence intervals (red shading) and the fit parameters were estimated using an iteration algorithm.

The data were well fit by a simple logistic regression (Supporting Information Eq. S1) indicating that low bubble O_2_ concentrations coincided with low ebullition rates (*R* ^2^ = 0.82, Fig. 7). When gas concentrations within the bubble approached equilibrium with the air (i.e., ~ 21%), the gas flux rapidly increases through the *K*_*s*_ (28.6% O_2_) and approached *ϕ*_max_. Although the data fit well to this simple relationship, a lack of data during high flux periods reduces the confidence of this relationship to parameterize maximum gas fluxes and maximum bubble O_2_ concentration (i.e., 95% confidence intervals, Fig. 7) and this relationship is expected to be site‐ and condition‐specific.

## *Discussion*

The results of this study reveal that ebullition represents a significant flux of photosynthetic O_2_ from eelgrass meadows—a flux that is not captured by measurement techniques relying only on concentration measurements of dissolved O_2_. These results are consistent with other shallow photosynthetic systems where ebullition of O_2_ can represent a significant fraction of ecosystem O_2_ exchange (Koschorreck et al. 2017; Howard et al. 2018). The presented gas ebullition rates, O_2_ concentrations, δ^18^O compositions in the evolved gas, and the conditions of their production revealed a photosynthetic origin of bubble production and highly variable O_2_ concentrations due to constantly varying biological and physical conditions. Under high irradiance at slack low tide, ebullition can represent nearly half of the photosynthetic O_2_ flux from seagrass meadows. Importantly, in ecosystems where autotrophic biomass approaches the carrying capacity of the system, the high daily rates of net production that lead to bubble ebullition are balanced by nighttime respiration. Therefore, the unquantified O_2_ flux via bubble ebullition can be a critical component for achieving measurement closure with model estimates of daily metabolic balance needed to determine the potential for seagrasses to persist and serve as blue carbon sinks (Koweek et al. 2018).

The highest daily oxygen ebullition observed in this study (up to 11 mmol O_2_ m^−2^ d^−1^) is consistent with ebullition rates estimated from temperate marsh ponds (up to 8 mmol O_2_ m^−2^ d^−1^, Howard et al. 2018) and directly measured in eutrophic lakes (5 mmol O_2_ m^−2^ d^−1^, Koschorreck et al. 2017) and shallow permeable sands (7 mmol O_2_ m^−2^ d^−1^, Cheng et al. 2014). Previous measurements of seagrass O_2_ flux conducted at this site, determined from dissolved O_2_ eddy covariance, report summer gross primary production rates (~ 2.7–16.6 mmol O_2_ m^−2^ h^−1^; Hume et al. 2011, Rheuban et al. 2014) which, on the low end, are similar to the maximal hourly O_2_ ebullition flux of 3.0 mmol O_2_ m^−2^ h^−1^ at the South Bay site. Eddy covariance measurements conducted at the same time at South Bay measured gross primary production rates of 3.9 ± 0.7 mmol O_2_ m^−2^ h^−1^ (Long et al. 2019), which are very similar to the presented maximal hourly rates of bubble ebullition. This suggests that during periods conducive to bubble formation, O_2_ ebullition has the potential to represent a large and generally unquantified fraction of seagrass photosynthesis. Indeed, the large range of fluxes determined in earlier studies could be partially due to the inability to capture O_2_ ebullition rates with dissolved O_2_ measurements. Most importantly, methods relying on dissolved oxygen measurements determined the sites to be either net heterotrophic (−24 ± 4 mmol O_2_ m^−2^ d^−1^, Rheuban et al. 2014), net autotrophic (19 ± 9 mmol O_2_ m^−2^ d^−1^, Hume et al. 2011), or in balance (2 ± 3 mmol O_2_ m^−2^ d^−1^, Long et al. 2019) during the summer time, indicating that bubble ebullition (~ 11 mmol O_2_ m^−2^ d^−1^) can be significant in determining the trophic status of shallow marine ecosystems.

The fraction of the photosynthetically derived O_2_ stored or transported in bubbles is not included when metabolic rates are derived solely from changes in dissolved O_2_ concentration. Therefore, the resulting low fluxes that occur frequently during mid‐day at low tide may be misinterpreted as photoinhibition or photorespiration effects (Ramus and Rosenberg 1980; Kosinski 1984; Hanelt 1996; Silva and Santos 2003). Neglecting bubble ebullition in field measurements may also help explain why short‐term laboratory measures of seagrass leaf photosynthesis vs. irradiance (performed in well‐mixed chambers at O_2_ concentrations below saturation to prevent bubble formation) often show no evidence of photoinhibition at irradiances reported to induce photoinhibition in the field (Mazzella and Alberte 1986; Zimmerman et al. 1991; Touchette and Burkholder 2000). Furthermore, the short‐term storage of O_2_ within bubbles trapped on leaf surfaces during brief periods of ideal bubble formation may be followed by redissolution as the dissolved O_2_ concentration decreases. This may produce a lag in measured dissolved O_2_ flux that is misinterpreted as a late afternoon recovery of photosynthetic capacity following the mid‐day depression. Similarly, changes in other physical parameters such as temperature, salinity, or tidal fronts with different dissolved O_2_ concentration could lead to dissolution of gas bubbles, resulting in dissolved O_2_ changes that are incorrectly attributed to biological processes such as photoinhibition or photorespiration.

Photosynthetic O_2_ production is responsible for creating the condition of gas supersaturation that leads to the formation of bubbles, but molecular diffusion across the bubble interface also acts to equilibrate the gas composition inside the newly formed bubble with that of the surrounding seawater. Consequently, O_2_ never represented more than ~ 40% of the entire gas volume because the bubble composition depends on the partial pressures of all of the dissolved gases in the surrounding seawater, which largely include dissolved N_2_, O_2_, and CO_2_ that diffuse into the bubble. Consistent with these multiple sources of gas to the bubbles, the fraction of the bubble gas with a photosynthetic origin, determined from measurements of δ^18^O (which clearly reflects these two sources of gas into the bubbles), suggests that photosynthetically derived O_2_ only accounts for up to 39% of the O_2_ in the captured bubbles during periods when ebullition was highest. Bubbles produced at night may represent the delayed dislodgement of bubbles produced on seagrass leaves during the daytime, in addition to bubbles of methane, nitrous oxide, carbon dioxide, or other trace gases released from the underlying anoxic sediments where they have been produced by microbial processes (Martens and Klump 1980; Huttunen et al. 2001; Gao et al. 2013). During nighttime bubble ebullition, the lower O_2_ concentrations in the bubbles are likely the result of increased time for equilibration with the lower dissolved O_2_ concentrations observed at night (~ 50–75% saturation), or possibly that sediment‐derived gas bubbles strip O_2_ out of the water column during their transport through the water column (Koschorreck et al. 2017).

The occurrence of ebullition depends on a number of biological and physical parameters whose interactions are not well characterized. At both sites, bubble ebullition over several days varied from zero to rates comparable to the gross primary production of seagrasses (Hume et al. 2011; Rheuban et al. 2014; Long et al. 2019). Bubble formation was observed to occur at low tide during the daytime when the difference between the internal seagrass aerenchyma gas pressures and the water pressure would increase due to the decreasing water depth and active photosynthesis (Larkum et al. 1989; Borum et al. 2007). While rates were maximal when low tide was coincident with high irradiance, ebullition rates were also dependent on the time of day since the diel maxima of O_2_ saturation and temperature were during the afternoon. Further complicating the attribution is the ~ 1.5–2 h time lag between these ideal conditions for bubble formation and their subsequent release from the leaf surface, which is triggered by bubble size, adherence to the leaf, and current and wave driven turbulence that can act to dislodge bubbles. Using tidally driven depth change (d*z*/d*t*) as an analog for flow velocity may explain why the largest fluxes were observed as flow increased following low tide, likely enhanced by current movement of the seagrass leaves that overcame the bubble adhesion to the leaves. However, increasing depth also leads to higher hydrostatic pressure and therefore reduced bubble size and buoyancy. These observations indicate that there is likely to be a dynamic relationship between increasing hydrostatic pressure, flow velocity, and bubble transport.

The logistic regression illustrates the relationship between gas production rates and gas concentrations that include a number of site‐specific factors that influence the production of bubbles including water O_2_ saturation, gas concentration gradients between the plant and the bubble, gas diffusion rates from the water into the bubble (and vice versa), hydrostatic pressure, and the volume at which the bubble becomes buoyant and detaches from the leaf. During periods of highest ebullition, the bubble gas still contains a majority of other gases (i.e., O_2_ < 41% [approx.]) and indicates that the bubble growth rate on the leaf surface over time (~ 1.5–2 h) facilitates gas diffusion into the bubbles, prior to ebullition to the atmosphere. The majority of the ebullition, which occurred during periods of high O_2_ saturation and irradiance, were consistent with previous field observations (Zieman 1974; Drifmeyer 1980; Hargraves 1982; Roberts and Caperon 1986) and indicate the importance of ebullition to ecosystem O_2_ budgets (Koschorreck et al. 2017; Howard et al. 2018). These results clearly show that bubble ebullition represents an important flux of photosynthetic O_2_ and presents key insights into the physical and biological dynamics of metabolism in highly productive seagrass ecosystems.

## Conflict of Interest

None declared.

## Supporting information


**Appendix S1**: Supplementary Material
