# Supplementary material for: Ebullition of oxygen from seagrasses under supersaturated conditions
Source: Limnol Oceanogr. 2019 Aug 8;65(2):314–24. doi: 10.1002/lno.11299 (PMC7043355; doi:10.1002/lno.11299)
Supplement: Supplementary file 1 — Appendix S1: Supplementary Material [file LNO-65-314-s001.pdf]

Supplemental Information

For: *Long MH, Sutherland K, Wankel SD, Burdige DJ, Zimmerman RC. (2019) Ebullition of Oxygen from Seagrasses under Supersaturated Conditions. Limnology and Oceanography.*

Data for this project can be found at the Biological and Chemical Oceanography Data Management Office (BCO-DMO) at: https://www.bco-dmo.org/award/710233

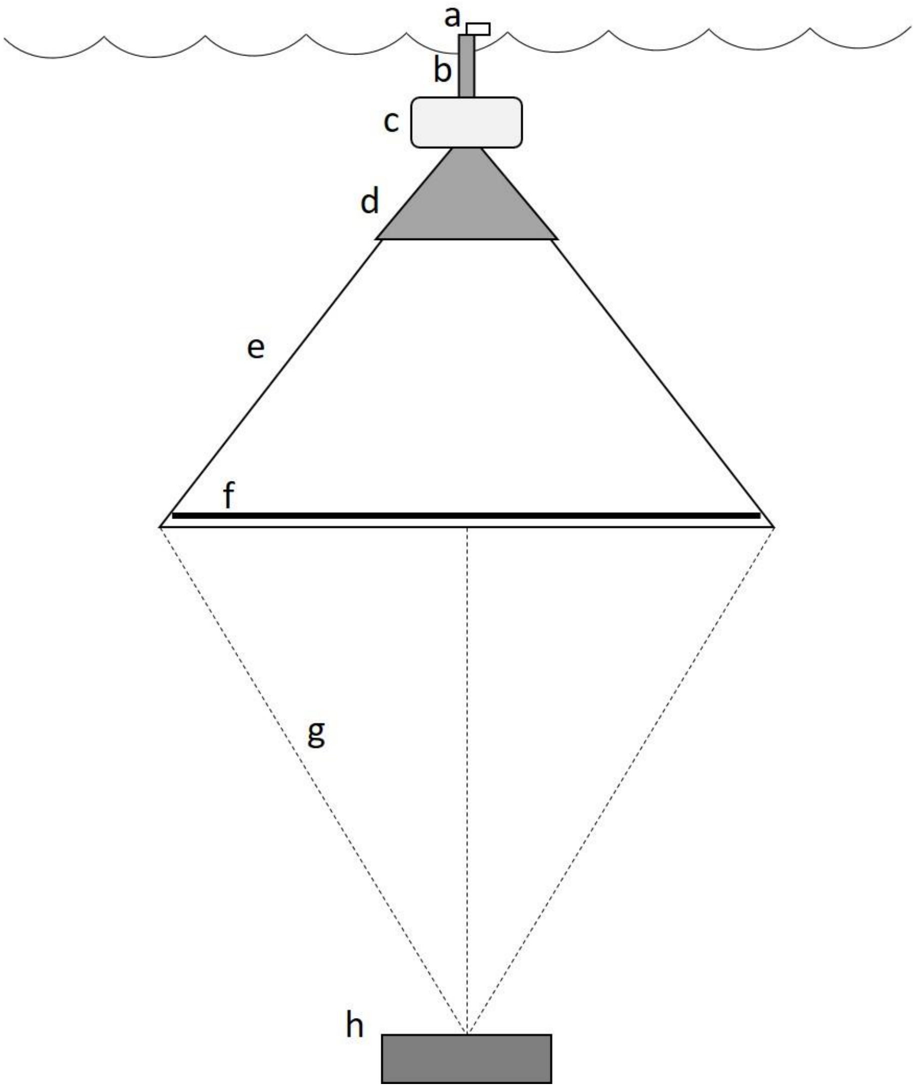

Figure S1. Schematic of bubble trap. The bubble traps consisted of a stockock for gas sampling (a) connected to a 30 mL syringe body (b) securely fastened to a laboratory funnel (d) that was provided buoyancy by a float (c). A clear, vinyl skirt was secured inside the funnel (e) whose circular shape was maintained by a steel ring (f) that was anchored to the bottom via lines (g) to a weight on the seafloor (h).

Table S1. Ebullition deployments, rates, and oxygen characteristics

Date Time	Site	Traps	Deployment Length	Gas Flux	Gas samples	Optode	O_2:Ar	$\delta^{18}O$
m/dd/yyyy HH:mm		*n*	h	mMol m^{-2} h^{-1}	*n*	% O_2	% O_2	‰
7/14/2017 11:30	SCB	4	1.46	0.17 ± 0.21	1	0.0	-	-
7/14/2017 12:45	SCB	6	1.38	0.00	-	-	-	-
7/14/2017 13:23	SCB	8	0.75	0.00	-	-	-	-
7/15/2017 9:00	SCB	6	22.62	0.00 ± 0.00	3	4.1 ± 3.2	-	-
7/15/2017 13:10	SCB	6	2.37	0.00	-	-	-	-
7/15/2017 14:22	SCB	6	1.25	0.00	-	-	-	-
7/16/2017 9:30	SCB	8	23.17	0.01 ± 0.00	5	3.4 ± 2.5	-	-
7/16/2017 14:00	SB	8	1.00	0.00	-	-	-	-
7/17/2017 8:30	SB	7	20.83	0.05 ± 0.09	7	6.7 ± 8.2	-	-
7/17/2017 11:45	SB	8	1.42	0.00 ± 0.00	1	0.0	-	-
7/17/2017 12:56	SB	8	1.20	0.00	-	-	-	-
7/18/2017 9:00	SB	8	20.00	0.00	-	-	-	-
7/18/2017 11:30	SB	8	1.63	0.97 ± 0.40	8	23.5 ± 4.0	20.9 ± 2.7	24.1 ± 3.0
7/19/2017 10:47	SB	7	0.90	2.15 ± 0.81	7	25.6 ± 2.5	24.3 ± 2.5	20.3 ± 2.6
7/19/2017 11:45	SB	8	1.12	5.41 ± 0.65	8	30.5 ± 1.1	27.1 ± 1.2	17.7 ± 1.3
7/19/2017 13:00	SB	6	1.00	1.33 ± 0.14	-	-	-	-
7/20/2017 8:00	SB	8	20.00	0.01 ± 0.00	-	-	-	-
7/20/2017 10:45	SB	8	0.92	1.07 ± 0.35	4	21.6 ± 2.8	23.3 ± 0.9	19.2 ± 0.8
7/20/2017 11:38	SB	8	0.93	3.42 ± 0.74	4	28.1 ± 2.8	25.6 ± 1.0	17.4 ± 1.0
7/20/2017 12:38	SB	8	1.08	6.25 ± 0.67	4	37.0 ± 2.7	30.3 ± 0.4	15.2 ± 0.3
7/20/2017 13:38	SB	8	0.73	7.31 ± 2.60	-	-	-	-
7/21/2017 0:00	SB	7	21.27	0.06 ± 0.08	5	13.9 ± 4.3	-	-
7/21/2017 9:00	SCB	4	20.37	0.01 ± 0.01	3	4.1 ± 5.2	-	-
7/21/2017 11:55	SCB	4	1.08	0.14 ± 0.12	2	20.6 ± 6.6	24.3 ± 0.0	21.0 ± 0.0
7/21/2017 12:30	SB	5	5.61	3.10 ± 0.18	-	-	-	-
7/21/2017 12:55	SCB	4	0.98	1.03 ± 0.41	4	25.4 ± 1.7	24.2 ± 0.4	19.6 ± 0.7
7/21/2017 13:45	SCB	4	0.61	3.91 ± 1.33	4	29.4 ± 4.2	24.5 ± 0.5	19.7 ± 0.5
7/21/2017 14:23	SCB	4	0.57	4.47 ± 0.70	-	-	-	-

Sites are Spider Crab Bay (SCB) and South Bay (SB). The time is the mean deployment time of replicate traps. The n is the number of trap or gas sample replicates. The ± values are the standard deviations of the replicates. The % O_2 are from both the optode and the isotope ratio mass spectrometer.

Sources of error in trap measurements - A potential source of error for the bubble trap measurements may be related to gas diffusion out of the collected gas while in the trap. During the daytime, individual traps were sampled for gas across deployment periods of 0.5 to 1.9 hrs. Plotting the deployment period (excluding overnight samples, which would be biased due to long trap deployment periods, Table S1) versus the oxygen concentration resulted in no relationship between deployment time and the O_2 concentration of the collected gas (R^2 = 0.0014, with a slope not different from zero; F = 0.0049, p = 0.9445). While our data set is limited (n = 37) there is no evidence that diffusion out of the trap significantly influenced the presented results. Additional sources of error in these trap measurements involve the limited area that they integrate across (0.07m^2), the retention of small bubbles on the trap walls, limited precision of gas volume measurements, and the loss of bubbles via equilibration that are not captured in the floating traps.

Gas sample O_2 concentrations - The concentration of O_2 in the collected gas samples, determined by both an O_2 optode and isotope ratio mass spectrometer -derived O_2:Ar ratio, were

significantly correlated ($r^2 = 0.58$, $p < 0.0001$) and not different from a 1:1 relationship (1.16 ± 0.16; $t = 1.03$, $p = 0.31$) (Figure S2). However, the regression left 42% of the variation among samples unexplained, possibly reflecting repeated septa piercing and limited precision of gas-tight syringe volume measurements.

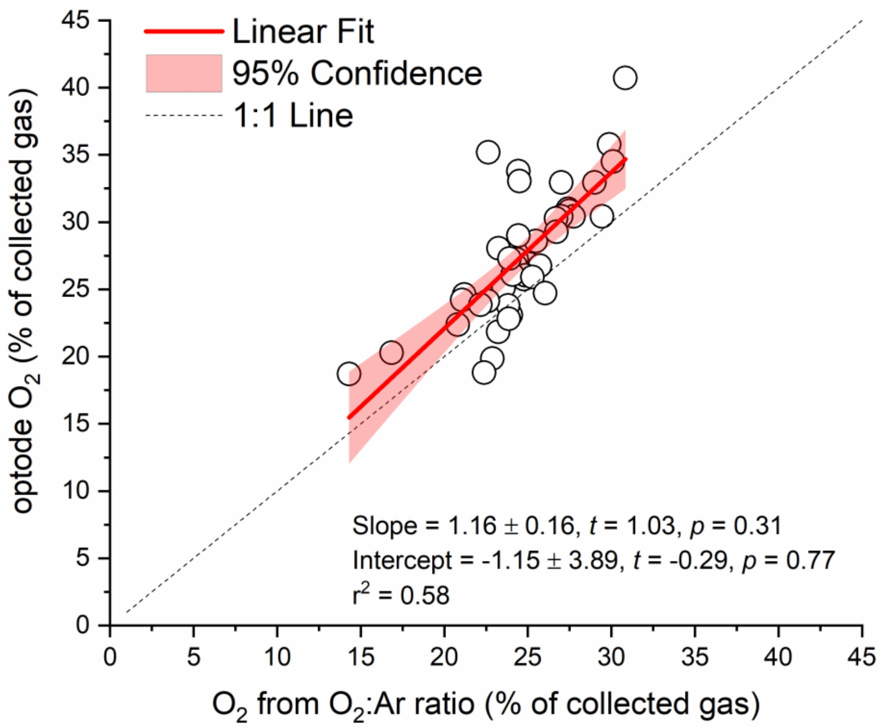

Figure S2. Correlation between O_2 percentages of collected gas from the O_2:Ar ratio of the sample (via isotope ratio mass spectrometer) relative to water at equilibrium and optode measurements. The linear correlation slope was not different from a 1:1 relationship (dashed, $t = 1.03$, $p = 0.31$) and the intercept was not different from zero ($t = -0.29$, $p = 0.77$). Analyses were conducted on the same sample for each analysis and deviation from the 1:1 relationship may be due to repeated vial septa piercing and limited precision of gas-tight syringe volume measurements.

Curve fitting - The polynomial fits of Figure 4 were fit with 2nd order polynomials in the form of $y = Ax^2 + Bx + C$. The constants for Figure 4a were A = 2.135 ± 1.616, B = -0.619 ± 2.752, and C = 0.008 ± 0.787. The constants for Figure 4b were A = 0.0006 ± 0.0005, B = -0.0720 ± 0.1097, and C = 1.654 ± 6.024.

A logistic function was used to illustrate the relationship between O_2 concentration and the gas fluxes in Figure 7 by: $\phi = \frac{\phi_{max}}{1+e^{-\lambda([O_2]-K_S)}}$ (Eq. S1), where ϕ is the measured gas flux (mmol gas m^{-2} h^{-1}), ϕ_{max} is the maximal gas flux, $[O_2]$ is the bubble O_2 gas concentration, K_s is the gas concentration that produces 50% of ϕ_{max}, and λ is the logistic gas flux rate (Figure 7). The equation was fit using a non-linear curve fitting function (OriginPro 2019) and a Levenberg Marquardt iteration algorithm where K_s, ϕ_{max}, and λ were initialized at a value of 1.0. A total of 18 iterations were performed until a X^2 tolerance value of 1.0×10^{-12} was reached.

Table S2. Correlation coefficients, *F* values, and *p* statistics for regressions of the gas fluxes and environmental parameters

	Time Lag	PAR			O_2			Depth			Temperature		
	(h)	*R*	*F*	*p*	*R*	*F*	*p*	*R*	*F*	*p*	*R*	*F*	*p*
**South Bay**	0.00	0.19	0.1	0.814	-0.03	0.0	0.974	0.76	2.7	0.243	0.75	2.5	0.255
	-0.25	0.92	10.7	0.082	0.38	0.3	0.619	0.62	1.2	0.383	0.84	4.8	0.160
	-0.50	0.95	19.2	0.048	0.65	1.4	0.352	0.37	0.3	0.626	0.90	8.9	0.097
	-0.75	0.96	23.7	0.040	0.70	2.0	0.297	-0.05	0.0	0.945	0.97	36.6	0.026
	-1.00	0.97	34.9	0.028	0.86	5.7	0.140	-0.55	0.9	0.449	1.00	1120.5	0.001
	-1.25	0.98	50.9	0.019	0.94	15.9	0.057	-0.81	3.9	0.186	1.00	300.3	0.003
	-1.50	0.99	66.9	0.015	1.00	778.9	0.001	-0.93	13.6	0.066	0.99	153.8	0.006
	-1.75	0.99	68.5	0.014	0.97	35.2	0.027	-0.98	53.0	0.018	0.99	88.2	0.011
	-2.00	1.00	288.5	0.003	0.97	31.3	0.031	-1.00	252.4	0.004	0.99	82.1	0.012
	-2.25	0.98	54.2	0.018	0.96	21.5	0.044	-1.00	234.8	0.004	0.99	95.8	0.010
	-2.50	0.96	25.0	0.038	0.95	18.1	0.051	-0.99	193.7	0.005	0.99	94.8	0.010
	-2.75	0.96	23.3	0.040	0.95	17.0	0.054	-1.00	227.7	0.004	0.99	93.8	0.010
	-3.00	0.93	13.5	0.067	0.95	19.2	0.048	-0.99	153.1	0.006	0.99	67.3	0.015
**Spider Crab Bay**	0.00	0.62	1.2	0.382	-0.37	0.3	0.632	0.89	7.3	0.114	0.44	0.5	0.556
	-0.25	0.68	1.8	0.317	0.11	0.0	0.888	0.78	3.2	0.216	0.62	1.2	0.383
	-0.50	0.86	5.6	0.143	0.57	1.0	0.431	0.57	1.0	0.432	0.90	8.1	0.105
	-0.75	0.89	7.6	0.110	0.77	2.8	0.235	0.13	0.0	0.871	0.95	17.0	0.054
	-1.00	0.91	9.5	0.091	0.87	6.0	0.134	-0.43	0.4	0.574	0.95	20.1	0.046
	-1.25	1.00	2586.7	0.000	0.95	19.7	0.047	-0.75	2.6	0.250	0.95	18.5	0.050
	-1.50	0.99	93.3	0.011	0.95	20.1	0.046	-0.88	6.8	0.121	0.95	17.5	0.053
	-1.75	0.96	24.3	0.039	0.97	29.4	0.032	-0.93	13.2	0.068	0.94	16.0	0.057
	-2.00	0.90	8.1	0.105	0.96	24.4	0.039	-0.94	16.1	0.057	0.94	16.2	0.057
	-2.25	0.94	15.8	0.058	0.96	24.1	0.039	-0.98	50.8	0.019	0.94	16.4	0.056
	-2.50	0.93	13.2	0.068	0.79	3.3	0.213	-0.95	20.2	0.046	0.96	24.0	0.039
	-2.75	0.96	26.0	0.036	0.80	3.5	0.202	-0.97	28.8	0.033	0.97	34.6	0.028
	-3.00	0.97	36.5	0.026	0.89	8.0	0.106	-0.97	27.5	0.035	0.97	38.3	0.025

Correlation coefficients (*R*), *F*, *p* values are for fits in Figure 6. Grey shading indicates significant relationships at $p < 0.060$.
